# β-lactam resistance associated with β-lactamase production and porin alteration in clinical isolates of *E*. *coli* and *K*. *pneumoniae*

**DOI:** 10.1371/journal.pone.0251594

**Published:** 2021-05-20

**Authors:** Sara M. Khalifa, Abeer M. Abd El-Aziz, Ramadan Hassan, Eman S. Abdelmegeed

**Affiliations:** Department of Microbiology and Immunology, Faculty of Pharmacy, Mansoura University, Mansoura, Egypt; Nitte University, INDIA

## Abstract

β-lactam resistance represents a worldwide problem and a serious challenge for antimicrobial treatment. Hence this research was conducted to recognize several mechanisms mediating β-lactam resistance in *E*. *coli* and *K*. *pneumoniae* clinical isolates collected from Mansoura University hospitals, Egypt. A total of 80 isolates, 45 *E*. *coli* and 35 *K*. *pneumoniae* isolates, were collected and their antibiotic susceptibility was determined by the Disc diffusion method followed by phenotypic and genotypic detection of extended-spectrum β-lactamases (ESBLs), AmpC β-lactamase, carbapenemase enzymes. The outer membrane protein porins of all isolates were analyzed and their genes were examined using gene amplification and sequencing. Also, the resistance to complement-mediated serum killing was estimated. A significant percentage of isolates (93.8%) were multidrug resistance and showed an elevated resistance to β-lactam antibiotics. The presence of either ESBL or AmpC enzymes was high among isolates (83.75%). Also, 60% of the isolated strains were carbapenemase producers. The most frequently detected gene of ESBL among all tested isolates was *bla*_CTX-M-15_ (86.3%) followed by *bla*_TEM-1_ (81.3%) and *bla*_SHV-1_ (35%) while *the Amp-*C gene was present in 83.75%. For carbapenemase-producing isolates, *bla*_NDM1_ was the most common (60%) followed by *bla*_VIM-1_ (35%) and *bla*_OXA-48_ (13.8%). Besides, 73.3% and 40% of *E*. *coli* and *K*. *pneumoniae* isolates respectively were serum resistant. Outer membrane protein analysis showed that 93.3% of *E*. *coli* and 95.7% of *K*. *pneumoniae* isolates lost their porins or showed modified porins. Furthermore, sequence analysis of tested porin genes in some isolates revealed the presence of frameshift mutations that produced truncated proteins of smaller size. β-lactam resistance in *K*. *pneumoniae* and *E*. *coli* isolates in our hospitals is due to a combination of β-lactamase activity and porin loss/alteration. Hence more restrictions should be applied on β-lactams usage to decrease the emergence of resistant strains.

## 1. Introduction

In the 21^st^century, the emergence and dissemination of resistant bacteria to antimicrobial agents is considered a challenging and threat to global public health as antibiotic resistance leads to higher medical costs, longer hospital stays, and increased mortality [1, 2]. β-lactam antibiotics are among the most commonly prescribed antibiotics due to their minimal side effects and broad antibacterial spectrum. However, various mechanisms are responsible for resistance to β-lactam compounds, such as the production of degrading enzymes, alteration of the drug target (modification of penicillin-binding proteins), decreased membrane permeability, and drug efflux pump [3, 4].

β-lactam compounds resistance is a rising problem and the production of different β-lactamases was documented to be the main cause of β-lactam resistance especially among gram-negative bacilli. Carbapenems group is often used as β-lactam of last resort in treating infections caused by multi-drug resistant bacteria especially those belonging to *Enterobacteriaceae*. However, in this family reduced susceptibility to carbapenem group can be frequently associated with carbapenemases production, membrane impermeability coupled with elevated expression of other β-lactamases, or a combination of these mechanisms [5–7].

The gram-negative bacteria outer membrane permits the passive diffusion of small hydrophilic solutes and important antibiotics (β-lactams and fluoroquinolones) by channel-forming proteins [8]. Porins are considered one class of these proteins that are present in gamma-proteobacteria members such as *E*. *coli*, *Salmonella*, *Shigella*, and others [9–12]. Porins also maintain the envelope integrity of the cells and act as receptors for bacteriocins and bacteriophages. Additionally, they participate in bacterial pathogenesis such as invasion, serum resistance, and adherence [13]. In gram-negative bacteria, different porins types have been identified and classified according to their functional structure, their activity, and their regulation and expression [10].

From the gram-negative bacteria, *E*. *coli* and *K*. *pneumoniae* which are responsible for the majority of nosocomial and community-acquired infections. The three major porins that have been identified in *E*. *coli* include OmpF, OmpC, and PhoE which differ from one another according to charge and size of solutes [10, 14, 15]. Also, *K*. *pneumoniae* contains two main porins, Ompk35, and Ompk36, through which hydrophilic solutes gain access to bacterial-cell [10]. Loss of membrane permeability may be due to mutation in porin that renders it non-functional or alteration in expression level or may be due to complete loss of the porin proteins [16]. Many studies illustrated that loss of Ompk35 and/or Ompk36 in *K*. *pneumoniae*, contribute to their resistance to cephalosporins and carbapenems [17–19]. Also, mutations in the *OmpC* or *OmpF* genes that result in subsequent porin loss have been reported in the resistance of Enterobacter spp., *E*. *coli*, and *S*. *marcescens*, for carbapenems [20, 21]. This work aimed to investigate the prevalence of porin alteration mediated resistance to β- lactam antibiotics in β-lactamase producing and non-producing strains of multidrug-resistant clinical isolates of *E*. *coli* and *K*. *pneumoniae*_._

## 2. Methods

### 2.1. Bacterial isolates

A total of 80 clinical isolates were separated from urine and sputum samples which were collected from microbiological laboratories in Mansoura University hospitals, Dakahlia governorate, Egypt. These isolates were identified as 45 isolates of *E*. *coli* and 35 isolates of *K*. *pneumoniae* using biochemical standard assay methods [22, 23]. This work was done after approval of the administrative authorities (Research Ethics Committee) in the faculty of pharmacy. Mansoura University, Egypt.

### 2.2. Antimicrobial susceptibility testing for isolates

The susceptibility of all isolates was determined using the disk diffusion method to different antimicrobials including cefotaxime (30 μg), ceftriaxone (30μg), ceftazidime (30 μg), cefepime (30 μg), piperacillin-tazobactam (36 μg), amoxicillin-clavulanic acid(30μg), imipenem (10 μg), amikacin (30 μg), trimethoprim/sulfamethoxazole (25 μg), Ofloxacin (5 μg), and nitrofurantoin (100 μg) on Mueller-Hinton agar plates [24]. All antibiotic discs were obtained from Oxoid, United Kingdom. According to zones of inhibitions, bacterial strains were classified as resistant, intermediate, or susceptible using Clinical and Laboratory Standard Institute guidelines [25].

### 2.3. Phenotypic screening for ESBLs enzymes production

The production of ESBL by clinical isolates was tested by Modified Double Disc Synergy Test (MDDST) using amoxicillin-clavulanic acid disc (20/10 μg) along with three third-generation cephalosporins discs {ceftazidime(30μg), ceftriaxone (30 μg) and cefotaxime (30 μg)} and one fourth-generation cephalosporin disc (cefepime 30 μg) [26]. The amoxicillin-clavulanate disc was centered on Mueller-Hinton agar plate lawned with test organism suspension equivalent to 0.5 McFarland standards. The discs of cephalosporins were placed around the amoxicillin-clavulanate disc with a distance of 15mm (for third-generation cephalosporins discs) and 20mm (for fourth-generation cephalosporin disc), and then the plates were incubated at 37°C. Positive results for ESBL production are considered by any increase in the inhibition zone around any of these cephalosporin discs towards the disc of amoxicillin-clavulanate.

### 2.4. Phenotypic screening for AmpC enzyme production

AmpC enzyme production for all isolated strains was determined by three- dimensional enzyme extract method [27]. Mueller Hinton agar plates were inoculated by *E*. *coli* DH5α and cefoxitin discs (30 μg) were centered on them. The extraction of crude enzymes from all isolates was done as mentioned by Livermore *et al*., 1984 [28]. A circular well and a linear slit were made in the agar plates as described before [29]. The enzyme extracts were added to the wells then the plates were kept upright for 5–10 min and incubated overnight at 37°C. Clear distortion of cefoxitin disc inhibition zone was shown in isolates which were AmpC β-lactamase producers while AmpC β-lactamase non-producers gave no distortion.

### 2.5. Phenotypic screening for carbapenemases enzymes production

All of the tested isolates were screened for carbapenemase production on Mueller-Hinton agar plates using the Modified Hodge test (MHT) as described by [30] using *E*. *coli* ATCC 25922 as an indicator and a meropenem disc (10μg).

### 2.6. PCR screening for β-lactamase genes and porin encoding genes

The presence of ESBL encoding genes (*bla*_TEM-1_, *bla*_SHV-1_, *bla*_CTX-M-15_), *Amp-c* gene, carbapenemase- encoding genes (*bla*_NDM-1,_ *bla*_VIM-1_, and *bla*_OXA-48_) among all *E*. *coli and K*. *pneumoniae* isolates and also porin encoding genes (*ompK35* for *K*. *pneumoniae* isolates and *ompC* and *ompF* for *E*. *coli* isolates) were examined by PCR utilizing the primers listed in Table 1. Genomic DNA was prepared from heating one-two colonies in 100 μl of distilled water at 95°C for 10 min. A reaction mixture contained 1μl of each primer (10 μM), 12.5 μl Dream Taq Green PCR Master Mix (2x) (Thermo Fisher Scientific Inc, USA), 1μl of total DNA, and 9.5 μl nuclease-free water. The PCR program was done as follows: initial denaturation at 94°C for 10 min; 35 cycles of DNA denaturation at 94°C for 30 s, annealing at a temperature specified for each primer as listed in Table 1 for 30 S and extension at 72°C for 1 min followed by final elongation step at 72°C for 7 min. The PCR products were visualized by electrophoresis in 1% agarose gels stained with ethidium bromide.

**Table 1:** Primers sequences used for screening tested genes.

Gene Type	Primer	Nucleotide sequence		Annealing Temp	Amplicon size (bp)	reference
Carbapenemase genes primers	VIM-1	F	5`–GAGCTCTTCTATCCTGGTG– 3`	52°C	103	[29]
		R	5`–CTTGACAACTCATGAACGG– 3`			
	NDM-1	F	5`–ACTTCCTATCTCGACATGC– 3`	52°C	133	
		R	5`–TGATCCAGTTGAGGATCTG– 3`			
	OXA-48	F	5`–TTGGTGGCATCGATTATCGG– 3`	55°C	743	
		R	5`–GAGCACTTCTTTTGTGATGGC– 3`			
AmpC genes primers	AmpC	F	5’-ACACGAGTTTGCATCGCCTG-3’	60°C	254	[27]
	AmpC	R	5’-CTGAACTTACCGCTAAACAGTGGAAT-3’			
ESBL genes primers	SHV	F	5’-ACTATCGCCAGCAGGATC-3’	53°C	356	[32]
	SHV	R	5’-ATCGTCCACCATCCACTG-3’			
	TEM	F	5’-GATCTCAACAGCGGTAAG-3’	50°C	786	
	TEM	R	5’-CAGTGAGGCACCTATCTC-3’			
	CTX-M-15	F	5’-GTGATACCACTTCACCTC-3’	49°C	255	
	CTX-M-15	R	5’-AGTAAGTGACCAGAATCAG-3’			
Porin primers	OmpC	F	TAG GTG CTT ATT TCG CCA TTC	56°C	1443	This study
	OmpC	R	GTA CGT GAT TAT CCT CAT GCG			
	OmpF	F	AGC ACT TTC ACG GTA GCG AAA	54°C	1341	
	OmpF	R	AGG CTG TTT TTG CAA GAC GTG			
	OmpK35	F	CGC TTT GGT GTA ATC GTT GTC	56°C	1128	
	OmpK35	R	GAC ACC AAA CTG TCA TCA ATG			

F, forward; R, reverse; bp, base pair.

### 2.7. Isolation and characterization of the outer-membrane proteins

The outer-membrane proteins (OMPs) of all *E*.*coli* and *K*. *pneumonia*e isolates were analyzed by SDS-PAGE [31] and compared to standard *E*.*coli* ATCC 25922 and *K*. *pneumoniae* ATCC 33495. Barwa and Shaaban procedure was used for the preparation of OMPs from all tested isolates [32]. Then OMPs were analyzed by SDS-PAGE (10%) and gels were visualized by staining with Coomassie blue.

### 2.8. Serum bactericidal assay

The resistance of all isolates to complement-mediated serum killing was estimated; bacteria were cultivated in nutrient broth until mid-logarithmic phase, centrifuged, washed twice with barbital buffer saline (BBS), and resuspended to a final concentration of 10^6^ CFU mL^-1^ in BBS. 40% normal human serum (NHS) in BBS was inoculated with the bacteria for 2 hrs at 37°C with shaking. Heat-inactivated NHS at 56°C for 30 min (HI-NHS) was considered as control. Samples which were taken at 0 and 2 hrs were serially diluted and then cell survival was determined by plating them onto nutrient agar plates followed by overnight incubation at 37°C. Each isolate was tested three times. The killing of bacteria by serum was estimated by measuring the decrease in the viable count over time [33].

### 2.9. Sequencing analysis of OMP genes

*OmpF* gene of three *E*. *coli* isolates (E7, E27, and E36), *ompC* gene of *one E*. *coli* isolate (E38) and *ompK35* gene of two *K*. *pneumoniae* isolates (K5 and K 23) were sequenced as previously described [34]. In brief, the tested outer membrane protein genes were amplified using PCR, each reaction mixture (50ul) contained 1 ng of DNA, 0.5 μm of each primer, 200 μm of dNTPs, 2mM MgCl_2_,10 μl of 5x Q5 buffer and 1 U of Q5 High-Fidelity DNA polymerase (NEB, UK). The QIA quick Gel Extraction kit (Qiagen, USA) was employed to purify PCR products according to the supplier’s protocol. The purified amplicons were subsequently sequenced in both directions with the Sanger method using the BigDye^®^ Terminator v3.1 Cycle Sequencing Kit and Applied Biosystems Genetic Analyzer 3500 (Thermo Fisher Scientific Inc, USA). The obtained sequence for each strain was analyzed and aligned with *ompF*, *ompC of E*. *coli* K12 reference gene sequence*s*, *and ompK35* reference gene sequence (GenBank Accession no JX310553.1) using Blast program of NCBI. To identify non-synonymous point mutations, DNA sequences were translated to amino acids using the BLAST software.

### 2.10. Statistical analysis

Data were fed to the computer and analyzed using IBM SPSS Corp. Released 2013. IBM SPSS Statistics for Windows, Version 22.0. Armonk, NY: IBM Corp. Qualitative data were described using number and percent. Significance of the obtained results was judged at the (0.05) level. Graph pad prism version 6.01 was used for figure design. Chi-Square test for comparison of 2 or more groups.

## 3. Results

### 3.1. Identification and antimicrobial susceptibility testing of bacterial strains

In this study, 80 clinical isolates of *Enterobacteriaceae* were collected from several microbiological laboratories in Mansoura University Hospitals. Of these, 45 isolates were identified as *E*. *coli* and 35 isolates were identified as *K*. *pneumoniae* using the standard biotyping method. Twenty- six *E*. *coli* isolates and 16 *K*. *pneumoniae* isolates were obtained from urine samples collected from the Urology and Nephrology Center whereas 5 and 6 isolates of *K*. *pneumoniae* were separated from urine samples collected from Emergency Hospital and Children Hospital respectively. Additionally, 19 *E*. *coli* isolates and 8 isolates of *K*. *pneumoniae* were obtained from sputum samples which were collected from Emergency Hospital and Children Hospital.

According to the breakpoints which were indicated in CLSI guidelines [25], the antibiotic susceptibility patterns for all isolates were analyzed. Resistance was observed to amoxicillin-clavulanic acid in 93.3% of *E*. *coli* isolates followed by 91.1% were resistant to ceftazidime, ceftriaxone, cefotaxime, ofloxacin, and trimethoprim-sulfamethoxazole in *E*. *coli* isolates. Also, *E*. *coli* strains showed a high resistance level to piperacillin-tazobactam (88.9%), cefepime (68.9℅), and imipenem (60%). In contrast, resistance to nitrofurantoin was seen only in 35.6% of *E*. *coli* isolates, and susceptibility to amikacin was retained by most of *E*. *coli* isolates (86.7%).

Furthermore, the highest resistance rate was recorded among *K*. *pneumoniae* isolates against ceftazidime (91.4%), ceftriaxone, amoxicillin-clavulanic acid, and trimethoprim-sulfamethoxazole (88.6℅), cefotaxime, and ofloxacin. (85.7℅), piperacillin-tazobactam and nitrofurantoin (82.9%), cefepime (74.3%), amikacin (67.7℅), and 23 isolates (65.7%) were resistant to imipenem.

### 3.2. Analysis of ESBLs and AmpC enzymes production

**Fig 1:**
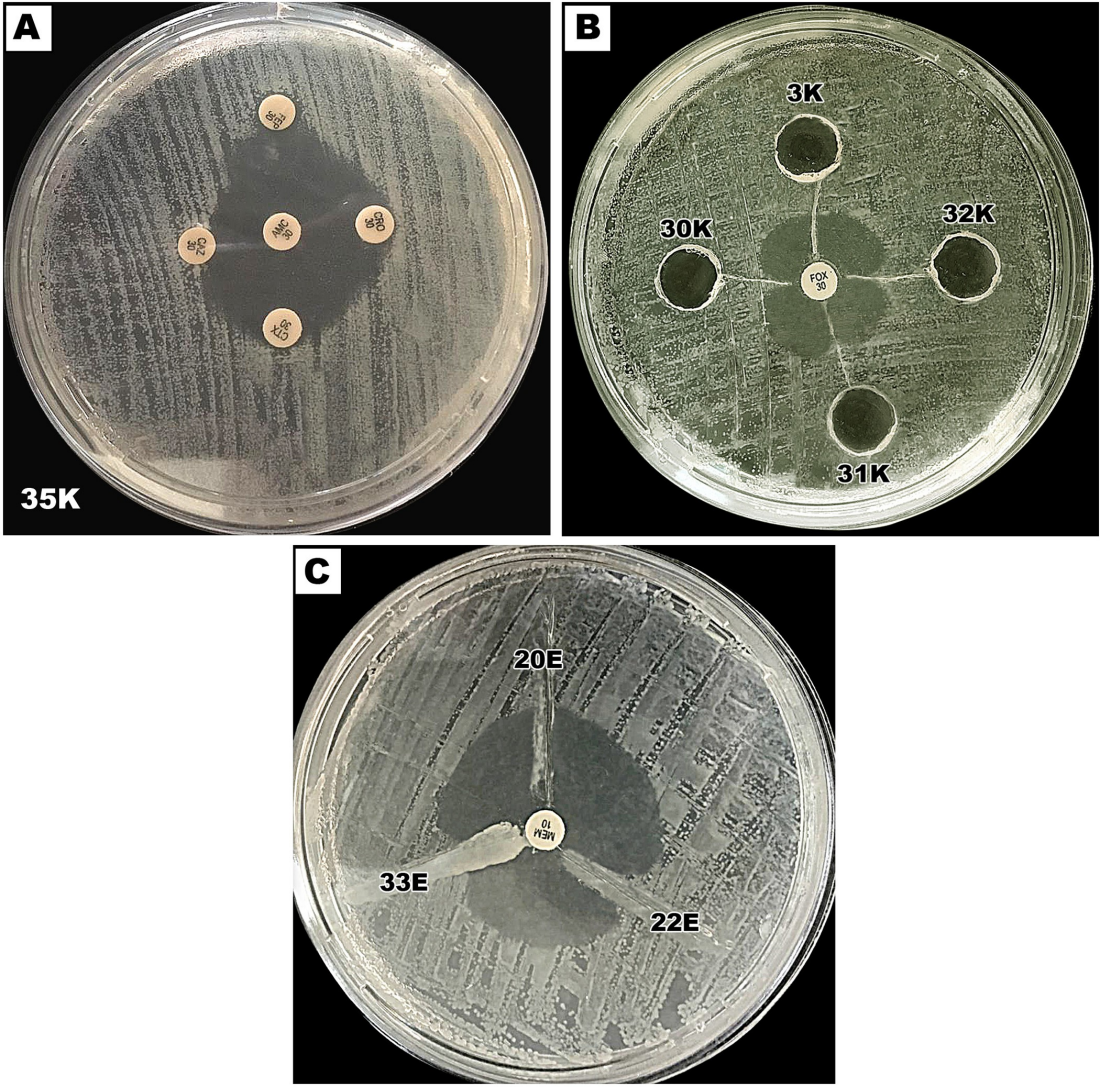
Phenotypic detection of β-lactamase production. **A.** Modified-double disc synergy test for determination of ESBL production. **B.** The 3-dimensional enzyme extract test for detection of AmpC production. **C.** Modified Hodge test for detection of carbapenemase production.

**Fig 2:**
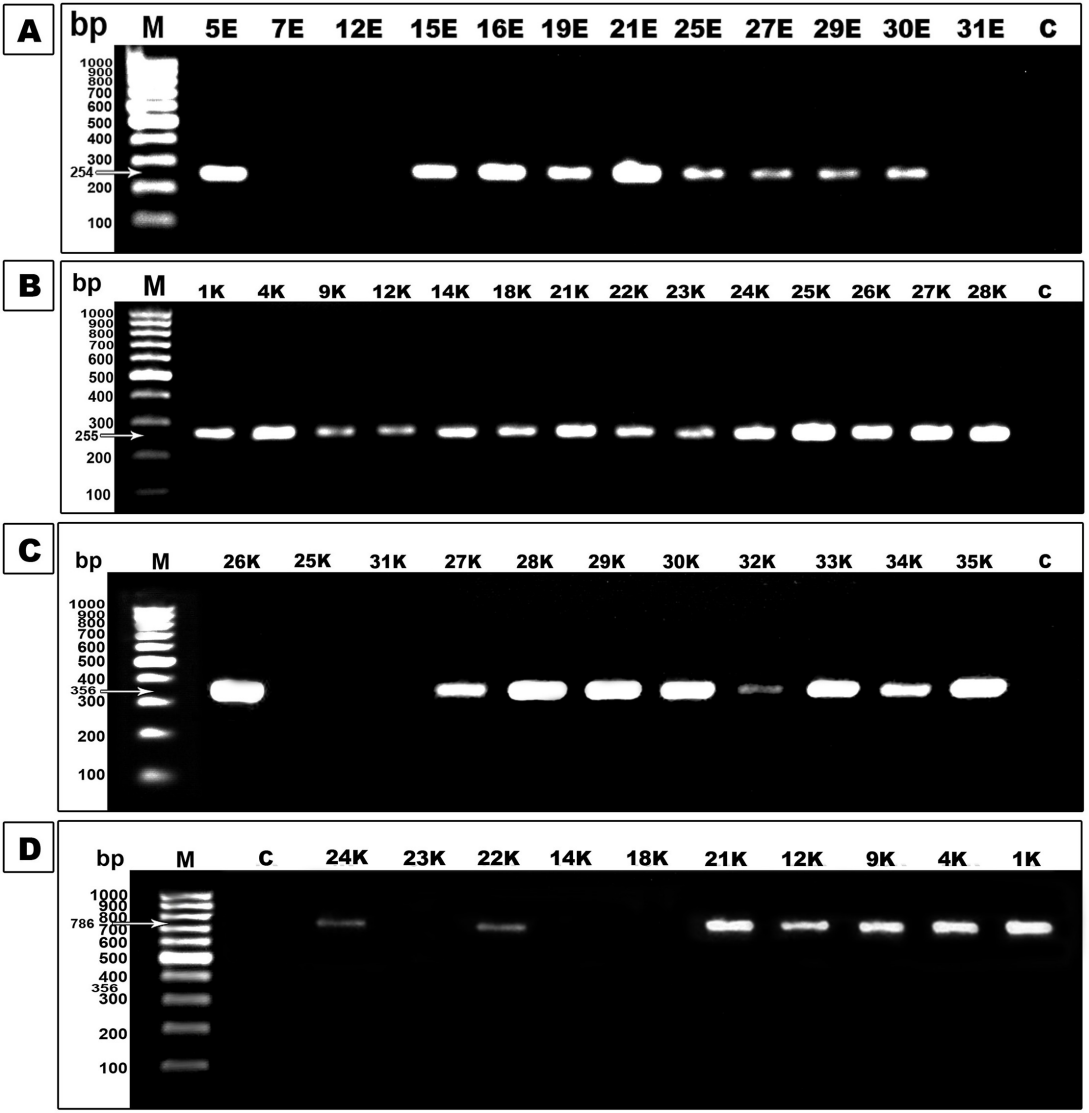
PCR assay for β-lactamase enzyme genes. Lane M was 100bp DNA marker and lane C was a negative control. **A.** Agarose gel electrophoresis of *AmpC* gene amplicons (254 bp). **B.** Agarose gel electrophoresis of *bla*_CTX-M-15_ gene amplicons (255 bp). **C.** Agarose gel electrophoresis of *bla*_SHV-1_ gene amplicons (356 bp). **D.** Agarose gel electrophoresis of *bla*_TEM-1_ gene amplicons (786 bp).

**Fig 3:**
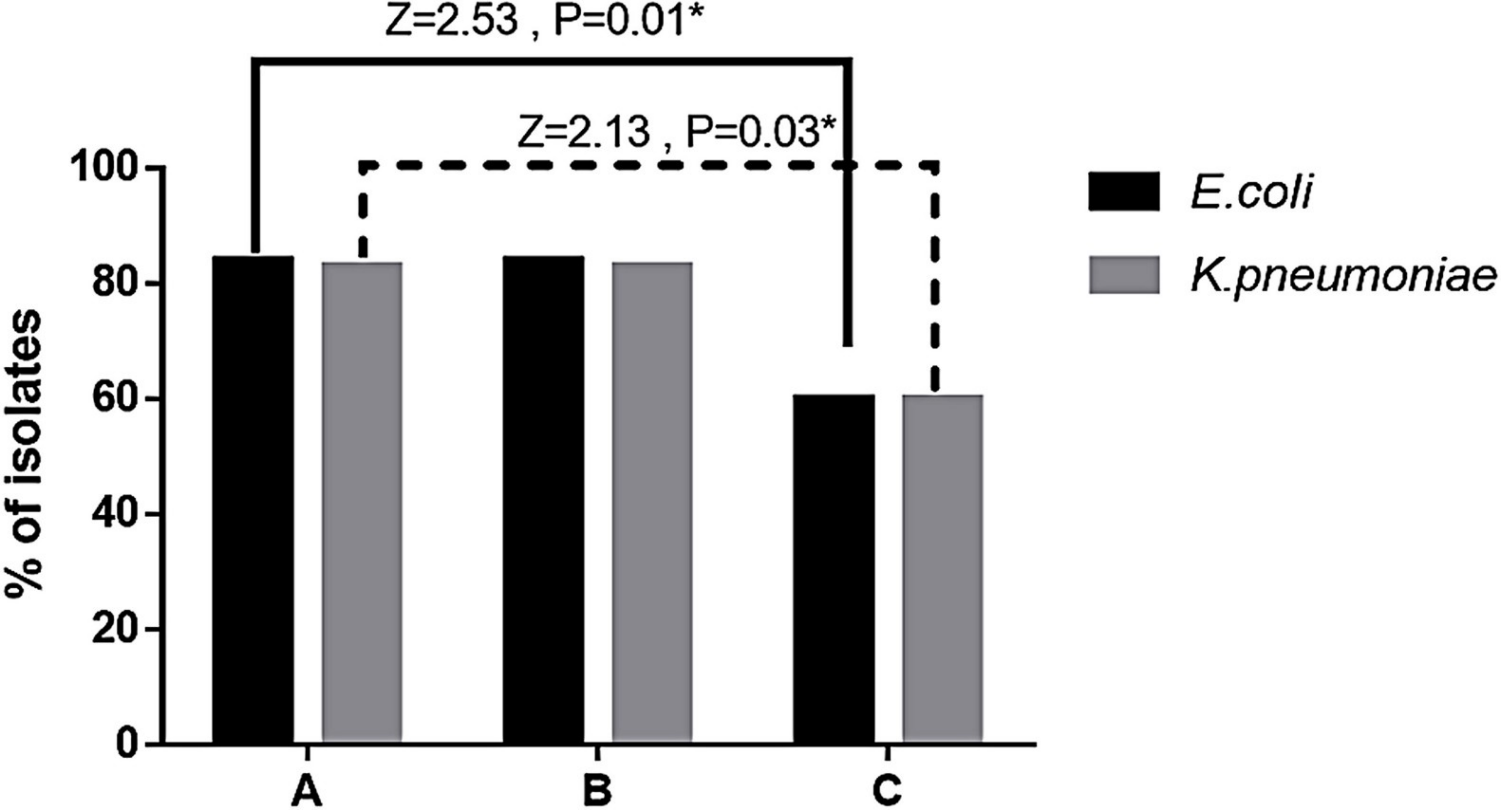
Frequency of β-lactamase producing tested isolates by phenotypic analysis. **A.** ESBL producers, **B.** AmpC producers, **C.** Carbapenemase producers. A statistically significant difference was detected between ESBL producers and carbapenemase producers among *K*. *pneumoniae* and similarly among *E*. *coli* without a significant difference between ESBL producers and AmpC producers in either type. Results were analyzed with Z-test for proportion with *p*<0.05*.

ESBLs and AmpC enzymes were both phenotypically (Fig 1) and genetically detected (Fig 2). The Determination of ESBL production by the MDDST method showed that 38 isolates of *E*. *coli* (84.4%) and 29 isolates of *K*. *pneumoniae* (82.9%) were considered positive for ESBL production (Figs 1A and 3). On the other hand, AmpC production which was detected by the three-dimensional extract method indicated that 38 *E*. *coli* isolates and 29 *K*. *pneumoniae* isolates were AmpC producers (Figs 1B and 3B). Moreover, phenotypic tests showed that 3 *E*. *coli* isolates and 6 *K*. *pneumoniae* isolates were considered ESBL and AmpC non-producers besides, 4 *E*. *coli* isolates produced only ESBL, and 4 *E*. *coli* isolates had AmpC activity only among all tested isolates while the remainder 34 *E*. *coli* produced both ESBL and AmpC enzymes.

**Fig 4:**
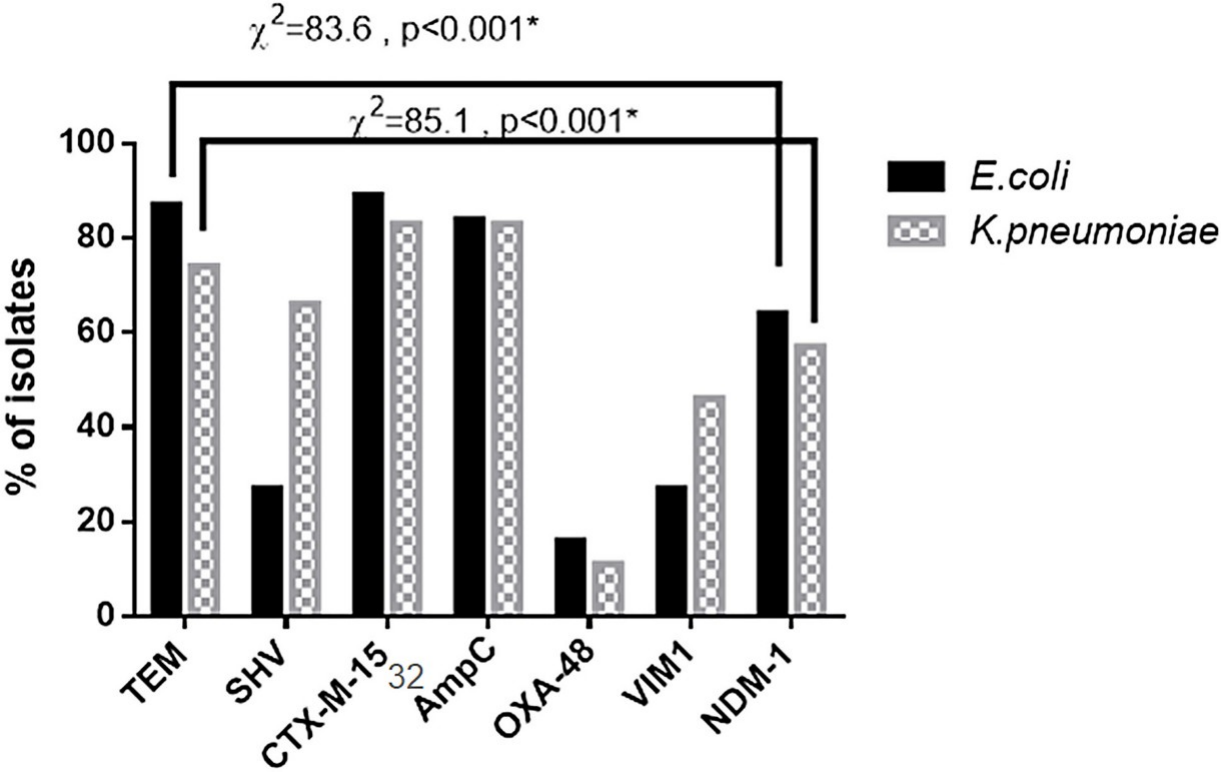
Percentage of ESBL, AmpC β-lactamase, and carbapenemase genes among the tested isolates. There is a statistically significant difference between different genes studied among *K*. *pneumoniae* and also among *E*. *coli* (*p*<0.001*). Results were analyzed with the Chi-Square test.

Considering PCR analysis, 95.6% of *E*. *coli* isolates (43/45) and 85.7% of *K*. *pneumoniae* (30/35) had at least one of the tested ESBL genes. The distribution of tested ESBL genes among the clinical isolates revealed that *bla*_TEM-1_and *bla*_CTX-M-15_were predominant among *E*. *coli* isolates (87% and 89% respectively). In contrast, *bla*_SHV-1_ was detected in 27% of *E*. *coli* isolates. Moreover, *bla*_SHV-1_, *bla*_TEM-1,_ and *bla*_CTX-M-15_ genes were predominantly carried by *K*. *pneumoniae* isolates whereas they were detected in 66%, 74%, and 86% (Fig 4). Also, *AmpC* gene detection by PCR revealed that it was harbored by 38 *E*. *coli* isolates and 29 *K*. *pneumoniae* isolates (Fig 4). The two *E*. *coli* isolates and also two *K*. *pneumoniae* which found to be sensitive to all tested antimicrobials showed that they did not harbor any of the tested β-lactamase genes.

### 3.3. Analysis of carbapenemases presence

The presence of carbapenemases was studied through the MHT test whereas 27 isolates of *E*. *coli* (60%) and 21 *K*. *pneumoniae* isolates (60%) had a positive Hodge test hence, they produced a distorted or clover-leaf shaped inhibition zone (Fig 1C). Also, it was found that 25 *E*. *coli* isolates and 21 *K*. *pneumoniae* isolates were positive for both ESBL and carbapenemase.

**Fig 5:**
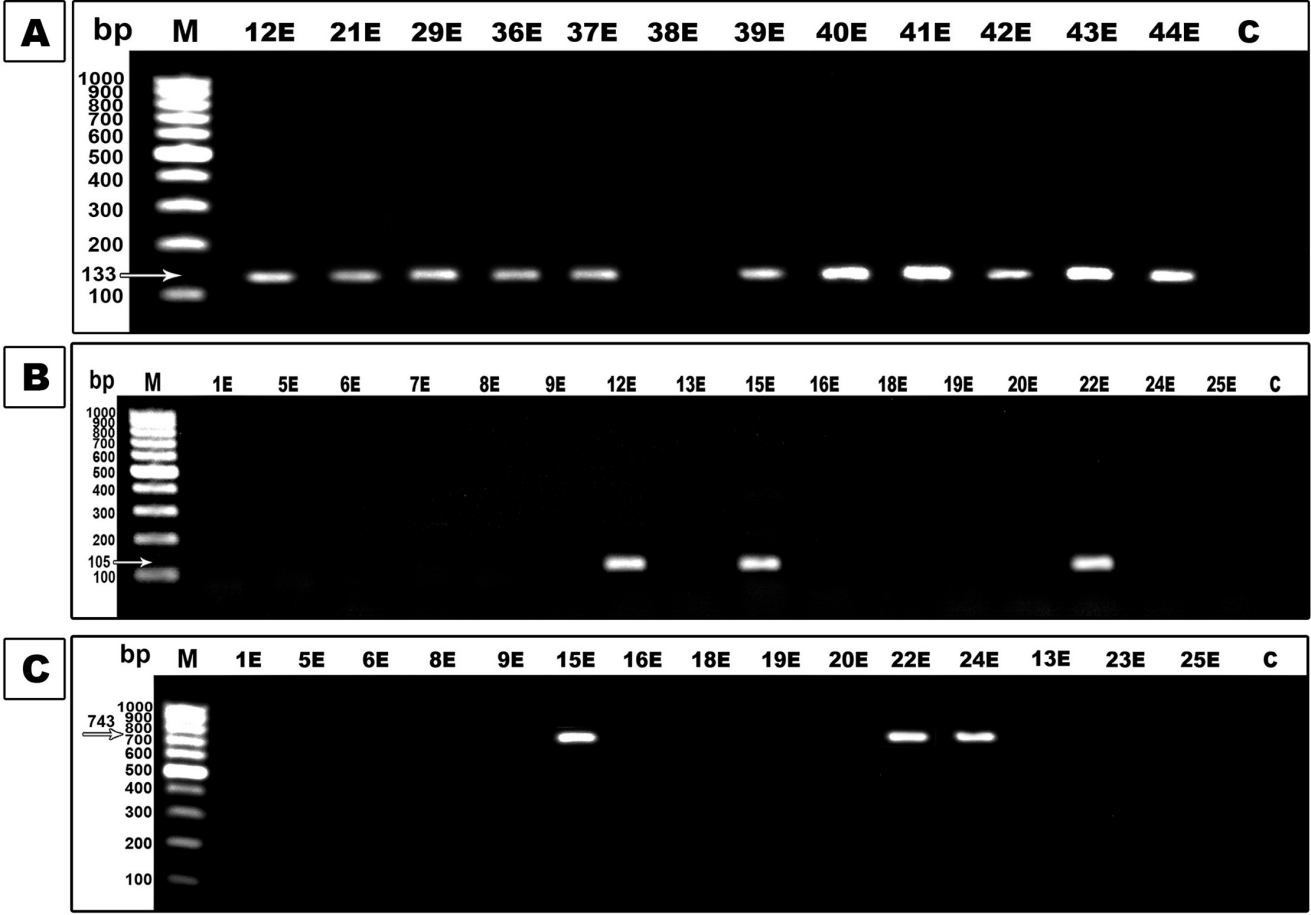
PCR assay for carbapenemases enzymes genes. Lane M was 100bp DNA marker and lane C was a negative control. **A.** Agarose gel electrophoresis of *the bla*_NDM-1_ gene amplicons (133 bp). **B.** Agarose gel electrophoresis of *bla*_VIM-1_ gene amplicons (105 bp). **C.** Agarose gel electrophoresis of *bla*_OXA-48_ gene amplicons (743 bp).

Also, PCR indicated that 29 *E*. *coli* strains and 19 *K*. *pneumoniae* strains harbored *the bla*_NDM-1_ gene, and 12 *E*. *coli* isolates and 16 *K*. *pneumoniae* isolates contained the *bla*_VIM-1_ gene (Fig 5). However, *bla*_OXA-48_ was present in only 7 isolates of *E*. *coli* and 4 isolates of *K*. *pneumoniae* (Fig 4). The results showed that the coexistence of the ESBL encoding gene and carbapenemase encoding gene in the same strain was found among all tested *E*. *coli* isolates. Also, 21 *K*. *pneumoniae* isolates were found to carry any of the tested carbapenemase genes but in association with ESBL and AmpC presence.

### 3.4. Analysis of outer-membrane proteins (porins)

**Fig 6:**
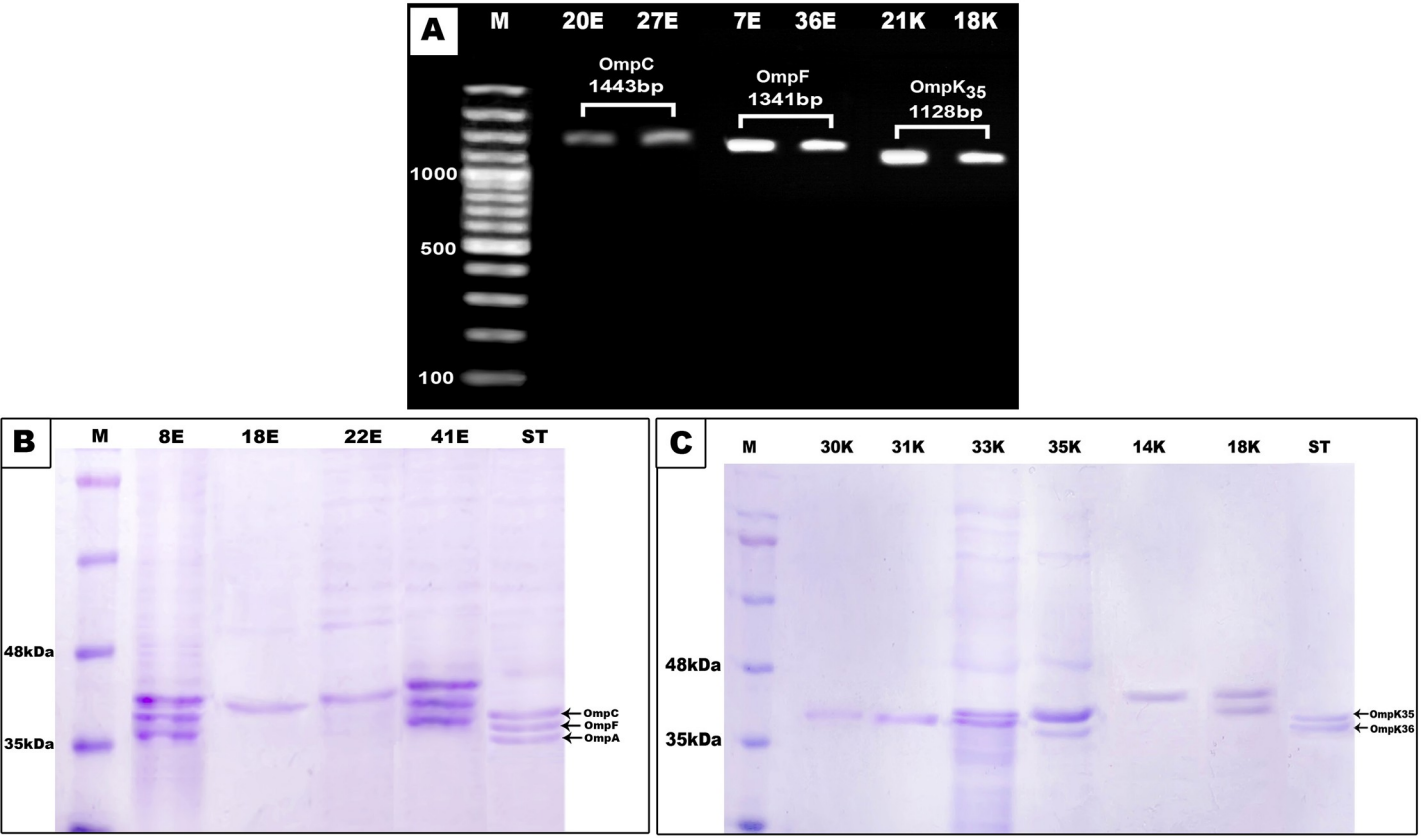
Outer-membrane porin protein and gene analyses. **A.** PCR detection of outer membrane porin encoding genes. Lane M: 100 bp plus DNA marker; lanes 1 and 2: amplified product of *OmpC* gene (1443 bp); lanes 3 and 4: amplified product of *OmpF* gene (1341 bp) which are porin genes in *E*. *coli*; lanes 5 and 6: amplified product of *OmpK35* gene (1128bp) which is porin gene in *K*. *pneumoniae*. **B.** SDS-PAGE analysis of outer membrane protein of some *E*. *coli* isolates. **C.** SDS-PAGE analysis of outer membrane protein of some *K*. *pneumoniae* isolates. Lane M: marker protein; lane ST: Standard strain used as control.

SDS-PAGE analysis of porins showed that 93.3% of *E*. *coli* isolates (42/45) and 85.7% of *K*. *pneumoniae* isolates (30/35) lost their porins or showed a modification in their electrophoretic migration pattern by comparison to the standard strain (Fig 6). From the results of porins analysis, it was found that 34 *E*. *coli* isolates that exhibited a loss in their porins had β-lactamase producing capability except 14 isolates had no carbapenemase-producing capability, 4 isolates did not produce AmpC enzyme and 4 isolates had no ESBL producing capability. Also, *E*. *coli* isolates with altered porin pattern had β- lactamase producing capability except for 6 isolates that could not produce carbapenemase enzymes, and 4 isolates were negative for ESBL enzymes but harbored one or more of the tested β-lactamase genes.

Regarding *K*. *pneumoniae*, 30 isolates that exhibited a loss in their porins or modification in porin pattern, had β -lactamase producing capability except for 8 isolates (no. 6, 15, 16, 20, 22, 25, 26, and 34) had no carbapenemases producing capability. Interestingly, 5 *K*. *pneumoniae* isolates that had unaltered porin pattern (isolates no. 2, 3, 13, 17, and 19) could not produce any of the tested β-lactamase enzymes.

Taking into account porin genes PCR analysis, among 45 *E*. *coli* isolates, 35 isolates exhibited *the OmpC* gene and 32 isolates had *the OmpF* gene. They had β-lactamase producing capability except 4 isolates did not produce AmpC or ESBL, 10 isolates did not produce carbapenemase, and 2 sensitive isolates which had no β-lactamase producing capability. On the other hand, 19 *K*. *pneumoniae* isolates harbored *the OmpK35* gene, as 14 of them produced ESBL and AmpC enzyme and 9 of them are considered also carbapenemase producers.

### 3.5. Serum bactericidal assay

The survival of bacteria in 40% NHS was measured for a period of 2 hrs. For *E*. *coli* isolates, 73.3% (33/45) were serum resistant and 90.9% (30/33) of these isolates were capable of producing at least one type of the tested β-lactamases (ESBL, AmpC and carbapenemases). On the other hand, 40% (14/35) of *K*. *pneumoniae* isolates were serum resistant from which 92.8% (13/14) were positive for all 3 tested β-lactamases. Furthermore, 28.5% (6/21) of serum sensitive *K*. *pneumoniae* isolates did not produce any type of the tested β-lactamases. Interestingly, we observed that 90.9% (30/33) of serum resistant *E*. *coli* isolates and 100% (14/14) of serum resistant *K*. *pneumoniae* isolates showed either loss or alteration in their porin pattern.

### 3.6. Sequencing analysis of OMP genes

**Fig 7:**
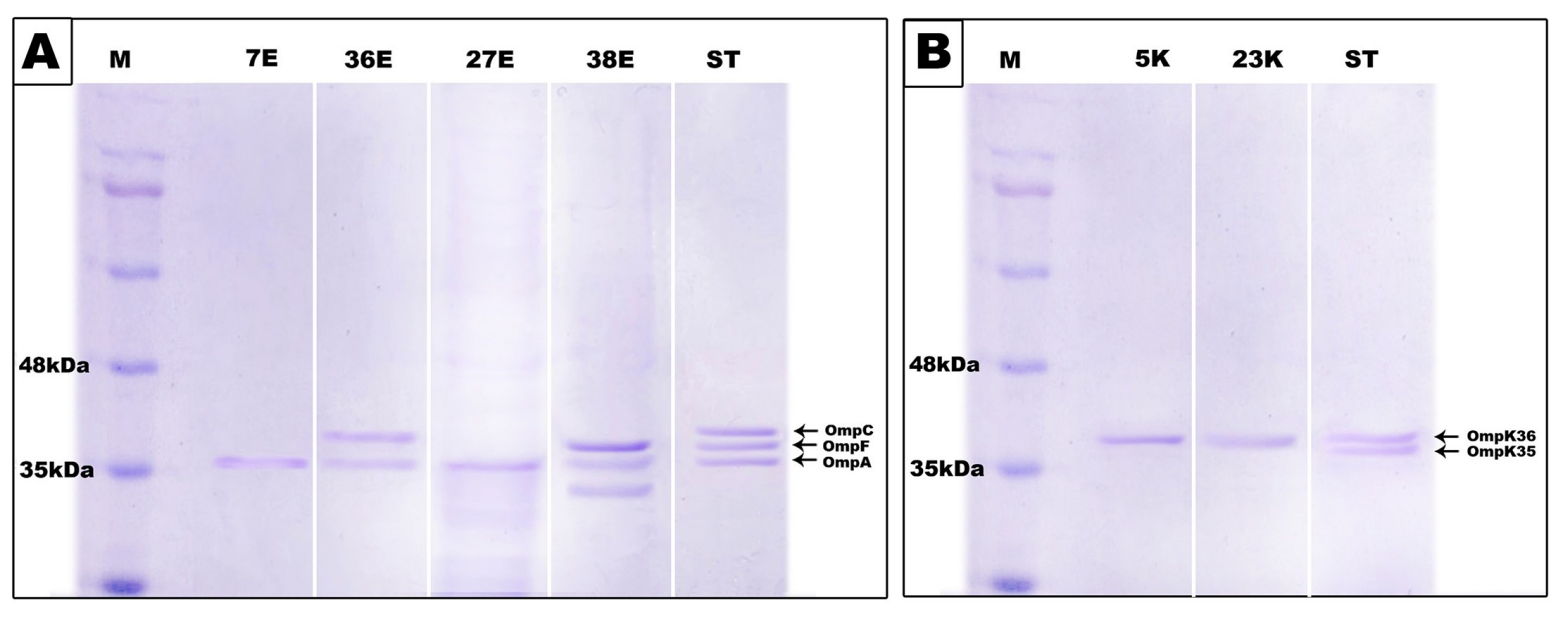
SDS-PAGE analysis of outer membrane proteins of sequenced genes. **A.** SDS-PAGE analysis of outer membrane protein of *E*. *coli* isolates 7E, 36E, 27E and 38E. **B.** SDS-PAGE analysis of outer membrane protein of *K*. *pneumoniae* isolates 5K and 23K. Lane M: marker protein; Lane ST: Standard strain used as control.

The sequence analysis of *ompF*, *ompC*, *and ompK35* genes of representative group of isolates (nos. 38E, 7E, 27E, 36E, 5K and 23K) demonstrated that most of these tested genes contained a frameshift mutation and all of them had a stop codon at different positions (Table 2). These mutations resulted in the production of truncated proteins of a smaller size than the wild type proteins. Hence, SDS-PAGE analysis of these isolates showed that they exhibited a modified porin pattern (Fig 7). *E*. *coli* isolate no.38 had a frameshift mutation and a stop codon at amino acid position 298 in *OmpC* porin and accordingly, a band of lower molecular mass (33 Kda) was observed (Fig 7)

**Table 2:** Detected mutations in tested *OmpF*, *OmpC and OmpK35* OMP genes.

Strain	Gene	Point mutation	Insertion or deletion of aa	Frameshift mutation
**38E**	***OmpC***	• **Fourteen missense mutations:** D49S, V50E, M57V, S85T, A86S, E90K, N91E, G138D, A274G, N275G, K276E, A277K, Q278H, N279K. • **Seven silent mutation** • **Stop codon aa 298**	**1 insertion: 88D**	**aa 280** (Deletion of one nucleotide at position 839–840 bp)
			**1 deletion: 173G**	
**7E**	***OmpF***	• **One missense mutations: K28Q** • **One silent mutation** • **Stop codon aa 67**	**None**	**aa 51** (Deletion of one nucleotide at position 150–151 bp)
**27E**		• **Two missense mutations: N26H, N51D** • **Two silent mutation** • **Stop codon aa 110**	**1 deletion: 2aa**	**aa 102** (insertion of one nucleotide at position 305 bp)
**36E**		• **Nine missense mutations: G48D, E51V, M60K, T61N, Y62L, A63C, R64P, L65S, G66W** • **Four silent mutation** • **Stop codon aa 68**	**None**	**None**
**5K**	***OmpK35***	• **One silent mutation** • **Stop codon aa 63**	**None**	**aa 62** (Deletion of one nucleotide at position 184–185 bp)
**23K**		• **Stop codon aa 16**	**None**	**aa 14** (Deletion of one nucleotide at position 39–40 bp)

## 4. Discussion

The widespread use of antibiotics leads to the emergence of many resistant microorganisms which is increasing worldwide and becoming one of the major serious health care problems [35, 36]. Our results showed a greater prevalence of resistance to common antibiotics and this agreed with that of Hassan *et al*. [37]. Indeed, the antibiotic susceptibility pattern of either isolated *E*. *coli* strains or *K*. *pneumoniae* strains revealed a high resistance rate (greater than 80%) which was recorded with ceftazidime, ceftriaxone, cefotaxime, amoxicillin/ clavulanic acid, trimethoprim/ sulfamethoxazole, piperacillin/ tazobactam, and ofloxacin, and these results are in agreement with other reports that showed higher resistance to sulfamethoxazole /trimethoprim (85.7%) and ciprofloxacin (72%) [38]. In contrast, Zaki *et al*. showed high susceptibility to sulfamethoxazole /trimethoprim and ciprofloxacin for *E*. *coli* isolates [39]. Here, it was found that 68.9% of *E*. *coli* isolates and 74.3% of *K*. *pneumoniae* isolates were resistant to cefepime and these findings were similar to those found in previous studies on antibiotic resistance in *E*. *coli* and *K*. *pneumoniae* [40, 41]. Carbapenems are considered the last shelter for gram-negative bacteria treatment but the development of carbapenem resistance is increasing in the Middle East. As the existing study demonstrated high resistance to imipenem in the tested *E*. *coli* isolates (60%) and *K*. *pneumoniae* (65.7%) and these findings were concordant with other studies that were performed in Egypt [39, 42, 43]. While high susceptibility of *E*. *coli* isolates was recorded with nitrofurantoin (64.4%) and amikacin (86.7%) but these antibiotics are less effective on *K*. *pneumoniae* isolates and this result was found to be similar to other studies [39, 42, 44].

β-lactam antibiotics are among the most widely prescribed drugs for bacterial infection treatment and resistance to them is an increasing problem [45]. The most prevalent mechanism of β-lactam resistance is the production of β-lactamase especially in *Enterobacteriaceae* [46]. Their continuous mutation leads to various β-lactamases enzymes [47]. Among them, ESBLs are of great interest and have been reported worldwide especially in *Enterobacteriaceae* [26, 48]. At present, more than 300 different ESBL variants have been identified [24] but the most common types which were reported in *Enterobacteriaceae* in several areas of the world are CTX-M, SHV, and TEM [49, 50]. Also, AmpC β-lactamases have been found all over the world and contribute to the problem of third generation cephalosporin resistance [51, 52]. Egypt is considered among the countries with the highest rate of ESBL production among *Enterobacteriaceae* [53], whereas previous studies in Egypt showed an elevated rate of ESBL producing isolates [39, 44, 54, 55]. This coincides with our results as ESBL production was detected in 38 isolates of *E*. *coli* (84.4%) and 29 isolates of *K*. *pneumoniae* (82.9%), while a low rate of ESBL production was observed by Abdelmegeed *et al*., in *E*. *coli* isolates [56]. As well as higher proportions were found in other countries including India (˃80%) and China (˃60%) [57]. PCR was used for confirmation of phenotypic outcomes and discrimination of different types of genes encoding ESBL enzymes among tested isolates. Many studies in Egypt and other countries showed that the most commonly detected β-lactamase–encoding gene among the ESBL-producing *Enterobacteriaceae* was *bla*_CTX-M_ [55, 58] whereas, the *bla*_CTX-M_ gene was able to be horizontally transferred by several mobile genetic elements [59]. Our data showed that *bla*_CTX-M-15_ was the most frequently observed ESBL-encoding gene (found among 89% of *E*. *coli* isolates and 86% of *K*. *pneumoniae* isolates) and this finding was consistent with the results of other studies performed in Egypt which indicated that *bla*_CTX-M-15_ was the most common ESBL encoding gene among ESBL-producing *Enterobacteriaceae* [55, 60, 61]. Besides, the next most frequently detected gene was *bla*_TEM-1_ which was found in 87% of *E*. *coli* isolates and 74% of *K*. *pneumoniae* isolates. Additionally, SHV was detected in 66% of *K*. *pneumoniae* isolates and a small percentage of *E*. *coli* isolates (27%) and these results are in agreement with a study of Abdallah *et al*., who indicated that CTX-M was the predominant detected ESBL gene followed by TEM then by SHV [55]. In contrast to our results, Yazdi *et al*., and Bajpai *et al*., reported that the most prevalent β-lactamase-encoding gene was *bla*_TEM_ followed by *bla*_SHV_ [62, 63] and in Zaki *et al*., study SHV was the most commonly detected gene (61.22%) [39]. The wider distribution of ESBL enzymes may be due to the mobilization of their genes on a genetic element as was reported by Shahid *et al*. [64].

Regarding AmpC enzyme production and the prevalence of its encoding gene, it was found in a higher percentage of *E*. *coli* and *K*. *pneumoniae* isolates (84.4% and 82.9% respectively). So the resistance to amoxicillin/clavulanate was high in tested isolates as AmpC enzymes are poorly inhibited by clavulanic acid [65]. In contrast, previous studies from Egypt showed a lower prevalence of the AmpC enzyme among *Enterobacteriaceae* [30, 56, 66]. The possible reason for the high prevalence of ESBL and AmpC producing organisms may be due to excessive usage of extended-spectrum cephalosporin in the treatment of gram-negative infection [67]. An important observation in this study is that many AmpC positive isolates were also ESBL-producer (67/80) and this result was equivocal with a study carried by Park *et al*., who indicated that the organism which was AmpC-producer is frequently associated with a high co-presence rate of ESBLs [67].

Carbapenems are the preferred therapy for ESBL producing *Enterobacteriaceae* [68]. However, the development of carbapenemases producing *Enterobacteriaceae* becomes a public health threat and leaves few therapeutic choices [69]. Many reports that have been published worldwide including Middle East countries on carbapenemases production among *Enterobacteriaceae* spp. showed a high presence of gram-negative bacteria which exhibited carbapenem resistance in different areas in Egypt [43, 70, 71]. Similarly in the current study, 60% of *E*. *coli* isolates and 60% of *K*. *pneumoniae* isolates were found to be carbapenemase producers and harbored carbapenemase encoding genes; *bla*_NDM-1_, *bla*_VIM-1,_ and *bla*_OXA-48_. This result is consistent with the high rate of carbapenemase production (69.8%) which was reported in Mahmoud *et al*. study [72] which may be due to widespread abuse of carbapenem in Egyptian hospitals [54, 73]. In contrast, Amjad *et al*. illustrated that 38% of *E*. *coli* isolates and 17% of *K*. *pneumoniae* were carbapenemase production positive [74], and Zaki *et al*., reported that 34.1% of *E*. *coli* isolates were carbapenemase producers [39]. Among the many types of carbapenemase encoding genes, the widespread of NDM type among *Enterobacteriaceae* has been reported in many countries [75–77]. The same was observed in our results which revealed that *bla*_NDM-1_ gene was the dominant carbapenemase encoding gene in tested isolates since it was present in all carbapenemase-producing isolates except E25 and E29 which did not contain any of the tested genes and K14 and K27 which contained *bla*_VIM-1_ and *bla*_OXA-48_ only respectively. These findings confirm previous reports which indicated that *bla*_NDM-1_ gene was common in Egypt and the Middle East. [43]. However, a low prevalence of *bla*_NDM-1_ gene was found in previous studies [66, 78] and no NDM-1 producing isolates were found in a study in China [79]. Besides this, *bla*_VIM-1_ was detected in 35% of the isolated strains while *bla*_OXA-48_ was detected in 13.8% of the tested isolates, whereas OXA-48 producing *Enterobacteriaceae* recently detected in Egypt [32, 39, 80]. Moreover, OXA-48 was commonly identified among *Enterobacteriaceae* in other countries including Saudi Arabia [81, 82]. North Africa, and Turkey [83]. Results of various studies on *K*. *pneumoniae* in Egypt are coincident with the current study as they reported that the NDM gene was the most predominant detectable carbapenemase gene followed by VIM [32, 84]. On the other hand, El kholy *et al* found that *bla*_OXA-48_ dominated (40.6%) followed by *bla*_NDM1_ (23.7%) among *K*. *pneumoniae* isolates and a small percentage of *E*. *coli* harbored *bla*_NDM-1_ [61]. It was reported that the NDM1 gene is harbored by diverse plasmids that also carry multiple resistance genes to macrolide, rifampin, carbapenem, cephalosporin, and Sulfamethoxazole and few treatment options are available for those strains which carried this gene [85]. So it was found in our study that the strains which harbored NDM-1 showed elevated variability resistance to various tested antibiotics. The current study showed excessive coexistence of various resistance genes in tested isolate and this leads to elevated variability in resistance and this agreed with that of Martin and Bachman [86].

The β-lactam resistance in gram-negative bacteria has been attributed not only to the presence of the hydrolyzing enzymes but also to the modification in the permeability of the outer membrane as well as to upregulation of multidrug efflux pump and alteration of antibiotic target proteins [11, 87]. Porin loss or mutation of the porin-coding sequence decreases membrane permeability and leads to impairment of antibiotic entry [88], hence loss of porins have been associated with a carbapenem and extended-spectrum cephalosporin resistance [89]. The mutational loss or alteration of porins in *Enterobacteriaceae* has been reported to be responsible for decreased susceptibility to cephalosporin and carbapenem [90, 91]. The increased resistance of ESBL producer *E*. *coli* and *K*. *pneumoniae* to cefotaxime and oxyimino β-lactam due to loss of porins was reported in many studies [92, 93]. Also, Domenech-Sanchez *et al*., found that the combination of decreased outer membrane permeability and the presence of SHV-1 and TEM-1 β-lactamase elevated cefotaxime MIC [94]. Therefore, we examined the porins as an additional resistance mechanism. Our results demonstrated that all the studied isolates showed porin loss or alteration in porin structure except three isolates of *E*. *coli* and 5 isolates of *K*. *pneumoniae*. And these results are in agreement with that obtained by Kitchel and coauthors who illustrated that the absence of OmpK35 was in 80% of *K*. *pneumoniae* isolates [95]. As well as it was observed that all of them harbored one ESBL and/or AmpC enzyme and this agreed with that of Wozniak *et al*. [88], additionally, this finding is consistent with that ESBL producing *K*. *pneumoniae* isolates don’t express OmpK35 and both OmpK35 and Ompk36 porins were expressed in *K*. *pneumoniae* isolate lacking ESBL which was reported in other studies [94, 96]. The loss of porins participates in the antimicrobial resistance of ESBL producing bacteria [16]. Besides, previous studies indicated that the absence or low expression of OmpK35in *K*. *pneumoniae* and OmpC and OmpF porins in *E*. *cloacae* has been associated with carbapenem resistance [34, 97]. The present data confirm this conclusion as almost all imipenem resistant isolates showed loss or alteration in tested porins and also the carbapenemase production was detected in them except 2 out of 23 strains of *K*. *pneumoniae* and 9 out of 27 strains of *E*. *coli* did not produce carbapenemase and this finding was concordant with the results of Barwa and Shaaban who found that all tested carbapenem-resistant isolates lost OmpK35 except one strain [32]. Besides that, it was reported that porin loss increases the resistance of ESBL-producing organisms to other non- β-lactam drugs such as fluoroquinolones [98, 99] and this explains the reason for the decreased susceptibility to ofloxacin by the tested organisms.

Since serum resistance is an important virulence trait for extraintestinal pathogenic *E*. *coli*, we reported that 73.3% of *E*. *coli* isolates collected from urine or sputum were serum resistant. However, the ability of serum sensitive isolates (26.7%) to cause extraintestinal infection may be due to the presence of antibodies that inhibited complement-mediated killing as indicated by Coggon *et al*. [100] In the current study, the association between serum resistance and production of different β-lactamases was clear among *E*. *coli and K*. *pneumoniae* isolates where the possession of β-lactamases was beneficial for some strains regarding serum survival and competitive fitness as reported elsewhere [101–104]. Several studies reported that certain porins in *E*. *coli* and *K*. *pneumonia* play a role in complement system activation as they promote the deposition of C1q and thus induce antibody-dependent classical pathway bactericidal activity. Therefore, loss or mutation of these immunogenic porins results in elevated resistance to complement-mediated killing [105–107]. This agreed with our results where 93.6% (44/47) of serum resistant *E*. *coli* and *K*. *pneumoniae* isolates showed either porin loss or alteration in their porin electrophoretic migration pattern.

Mutations in *ompK35* gene of tested *K*. *pneumoniae* isolates K5 and K23, *ompF* gene of *E*. *coli* isolates E7, E27, and E36 and *ompC* gene of *E*. *coli* isolate E38 involved a stop codon that formed truncated porins lacking the C-terminal phenylalanine residue which is essential for membrane anchoring [108] thus producing nonfunctional protein and porin cannot be inserted in the outer membrane. The same results were observed by Wozniak *et al*., who found a frameshift mutation and a stop codon at position 144 in the tested *ompK35* genes [88]. In addition, Wozniak *et al*., reported that OmpK36 was the preferred porin and was thus conserved by *K*. *pneumonia* because of its smaller pore than OmpK35 and was, therefore, more restrictive for antibiotic entrance. Hence, in our study, the band observed in the SDS-PAGE analysis of 5K and 23K (Fig 7) corresponds most likely to OmpK36.

The exceeding emergence of β-lactam resistance in *E*. *coli* and *K*. *pneumoniae* strains in Mansoura, Egypt is disturbing. Moreover, our results demonstrated that β-lactam resistance in *E*. *coli* and *K*. *pneumoniae* isolates were mediated by β-lactamases plus porin loss or mutation of the porin-coding sequence. Hence, the recommendation must be taken during the administration of β-lactam and carbapenem in our hospitals to decrease the spread of β -lactam resistant isolates.

## Supporting information

S1 Table Phenotypic and genotypic analysis of different β-lactamases in *E. coli* isolates. (DOCX)

S2 Table Phenotypic and genotypic analysis of different β-lactamases in *K. pneumonia* isolates. (DOCX)

S1 File (PDF)
