# Supplementary material for: β-lactam resistance associated with β-lactamase production and porin alteration in clinical isolates of E. coli and K. pneumoniae
Source: PLoS One. 2021 May 20;16(5):e0251594. doi: 10.1371/journal.pone.0251594 (PMC8136739; doi:10.1371/journal.pone.0251594)
Supplement: S1 Table — (DOCX) [file pone.0251594.s001.docx]

**S1 Table.** Phenotypic and genotypic analysis of different β-lactamases in *E. coli* isolates

| PCR analysis | | | | | | | Phenotypic test | | | Isolate |
| --- | --- | --- | --- | --- | --- | --- | --- | --- | --- | --- |
| *bla*_NDM-1_ | *bla*_VIM1_ | *bla*_OXA-48_ | *bla*_AmpC_ | *bla*_CTX-M-15_ | *bla*_SHV_ | *bla*_TEM_ | Hodge | AmpC | ESBL |  |
| + | + | - | + | + | - | + | + | + | - | 1E |
| + | + | - | + | + | + | + | + | + | + | 2E |
| - | - | - | - | + | - | - | - | - | + | 3E |
| + | + | - | - | + | - | + | + | - | + | 4E |
| + | + | - | + | + | + | + | + | + | + | 5E |
| - | - | - | - | + | - | - | - | - | + | 6E |
| + | + | - | - | + | - | + | + | - | + | 7E |
| + | + | - | + | + | + | + | + | + | + | 8E |
| + | + | - | + | - | - | + | + | + | + | 9E |
| + | - | - | + | + | - | + | + | + | + | 10E |
| - | - | - | + | + | - | + | - | + | + | 11E |
| + | - | - | + | + | - | + | + | + | + | 12E |
| - | - | - | + | + | - | + | - | + | + | 13E |
| + | - | - | + | + | - | + | + | + | + | 14E |
| + | + | + | + | + | - | + | + | + | + | 15E |
| + | - | - | + | + | - | + | + | + | + | 16E |
| - | - | - | + | - | - | + | - | + | + | 17E |
| - | - | - | + | + | + | + | - | + | + | 18E |
| + | + | - | + | + | - | + | + | + | + | 19E |
| - | - | - | + | + | - | - | - | + | - | 20E |
| + | - | + | + | + | - | + | + | + | + | 21E |
| + | + | + | + | + | - | + | + | + | + | 22E |
| + | - | - | + | + | - | + | + | + | + | 23E |
| + | + | + | + | + | - | + | + | + | + | 24E |
| - | - | - | + | + | + | + | + | + | + | 25E |
| - | - | - | + | + | - | + | - | + | + | 26E |
| - | - | - | + | + | - | + | - | + | + | 27E |
| + | - | - | - | + | - | - | - | - | - | 28E |
| - | - | - | + | + | - | + | + | + | + | 29E |
| - | - | - | + | + | - | + | - | + | + | 30E |
| + | - | + | + | + | + | + | + | + | + | 31E |
| - | - | - | + | + | - | + | - | + | + | 32E |
| + | - | - | + | + | - | + | - | + | + | 33E |
| - | - | - | - | - | - | - | - | - | - | 34E |
| - | - | - | - | - | - | - | - | - | - | 35E |
| + | - | - | + | + | - | + | + | + | + | 36E |
| + | - | + | + | + | + | + | + | + | + | 37E |
| - | - | - | + | + | - | + | - | + | + | 38E |
| + | - | - | + | + | - | + | + | + | - | 39E |
| + | - | - | + | + | + | + | - | + | + | 40E |
| + | - | - | + | + | + | + | + | + | + | 41E |
| + | - | - | + | - | + | + | - | + | - | 42E |
| + | - | - | + | + | + | + | + | + | + | 43E |
| + | - | - | + | + | + | + | + | + | + | 44E |
| + | + | + | + | + | - | + | + | + | + | 45E |
