# Supplementary material for: β-lactam resistance associated with β-lactamase production and porin alteration in clinical isolates of E. coli and K. pneumoniae
Source: PLoS One. 2021 May 20;16(5):e0251594. doi: 10.1371/journal.pone.0251594 (PMC8136739; doi:10.1371/journal.pone.0251594)
Supplement: S2 Table — (DOCX) [file pone.0251594.s002.docx]

**S2 Table**. Phenotypic and genotypic analysis of different β-lactamases in *K. pneumonia* isolates

| PCR analysis | | | | | | | Phenotypic test | | | Isolate |
| --- | --- | --- | --- | --- | --- | --- | --- | --- | --- | --- |
| *bla*_NDM-1_ | *bla*_VIM1_ | *bla*_OXA-48_ | *bla*_AmpC_ | *bla*_CTX-M-15_ | *bla*_SHV_ | *bla*_TEM_ | Hodge | AmpC | ESBL |  |
| _ | - | - | + | + | - | + | - | + | + | 1K |
| - | - | - | _ | - | - | - | - | - | - | 2K |
| - | - | - | - | - | - | - | - | - | - | 3K |
| + | + | - | + | + | + | + | + | + | + | 4K |
| + | - | - | + | + | + | + | + | + | + | 5K |
| - | - | - | + | + | + | + | - | + | + | 6K |
| + | + | - | + | + | + | + | + | + | + | 7K |
| + | - | - | + | + | + | + | + | + | + | 8K |
| + | + | - | + | + | + | + | + | + | + | 9K |
| + | + | - | + | + | - | + | + | + | + | 10K |
| + | + | - | + | + | + | + | + | + | + | 11K |
| + | + | - | + | + | - | + | + | + | + | 12K |
| - | - | - | - | - | - | - | - | - | - | 13K |
| - | + | - | + | + | + | - | + | + | + | 14K |
| - | - | - | + | + | - | + | - | + | + | 15K |
| - | - | - | + | + | + | + | - | + | + | 16K |
| - | - | - | - | - | - | - | - | - | - | 17K |
| + | + | - | + | + | + | - | + | + | + | 18K |
| - | - | - | - | - | - | - | - | - | - | 19K |
| - | - | - | - | - | - | - | - | - | - | 20K |
| + | + | - | + | + | + | + | + | + | + | 21K |
|  | - | - | + | + | + | + | - | + | + | 22K |
| + | - | - | + | + | + | - | + | + | + | 23K |
| + | + | - | + | + | + | + | + | + | + | 24K |
| - | - | - | + | + | - | + | - | + | + | 25K |
| - | - | - | + | + | + | + | - | + | + | 26K |
| - | - | + | + | + | + | + | + | + | + | 27K |
| + | + | - | + | + | + | + | + | + | + | 28K |
| + | + | - | + | + | + | + | + | + | + | 29K |
| + | - | - | + | + | - | + | + | + | + | 30K |
| + | + | + | + | + | + | + | + | + | + | 31K |
| + | + | + | + | + | + | + | + | + | + | 32K |
| + | + | - | + | + | + | + | + | + | + | 33K |
| - | - | - | + | + | + | + | - | + | + | 34K |
| + | + | + | + | + | + | + | + | + | + | 35K |
