# Supplementary figures and images for: β-lactam resistance associated with β-lactamase production and porin alteration in clinical isolates of E. coli and K. pneumoniae
Source: PLoS One. 2021 May 20;16(5):e0251594. doi: 10.1371/journal.pone.0251594 (PMC8136739; doi:10.1371/journal.pone.0251594)

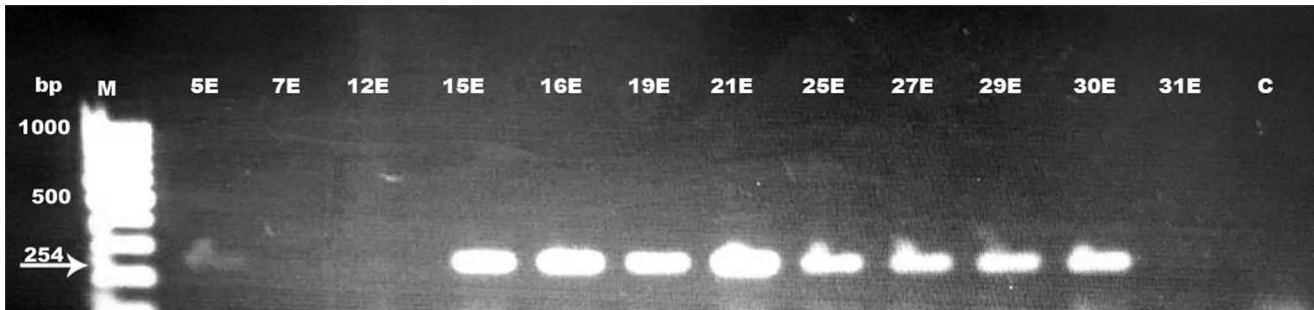

**Fig. (2) A**

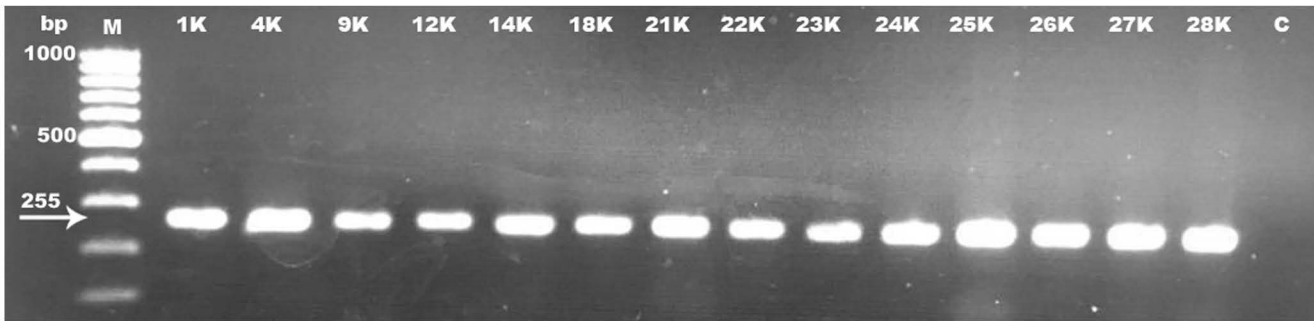

**Fig. (2) B**

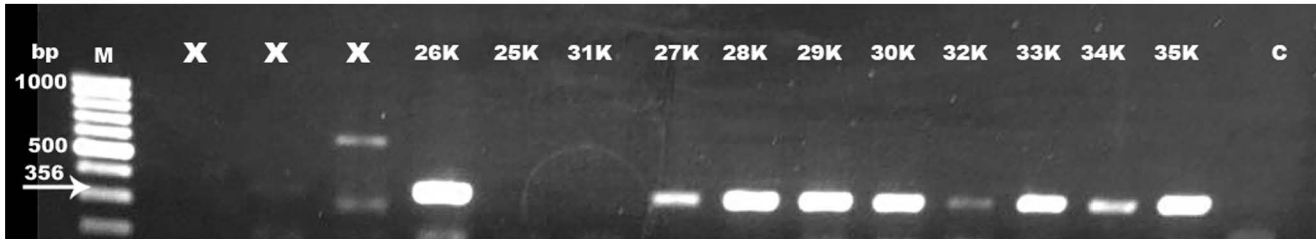

**Fig. (2) C**

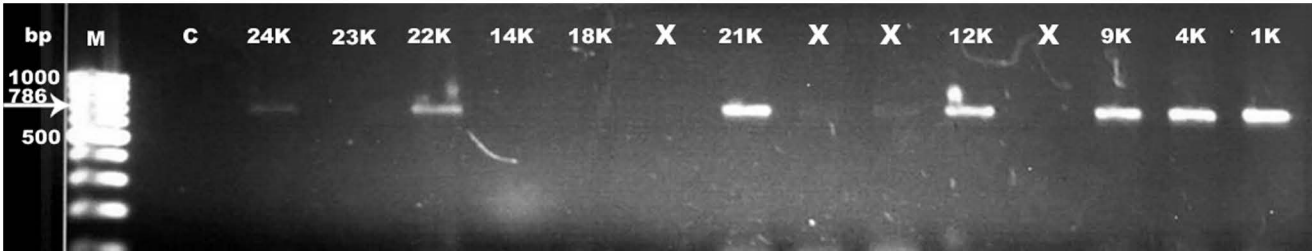

**Fig. (2) D**

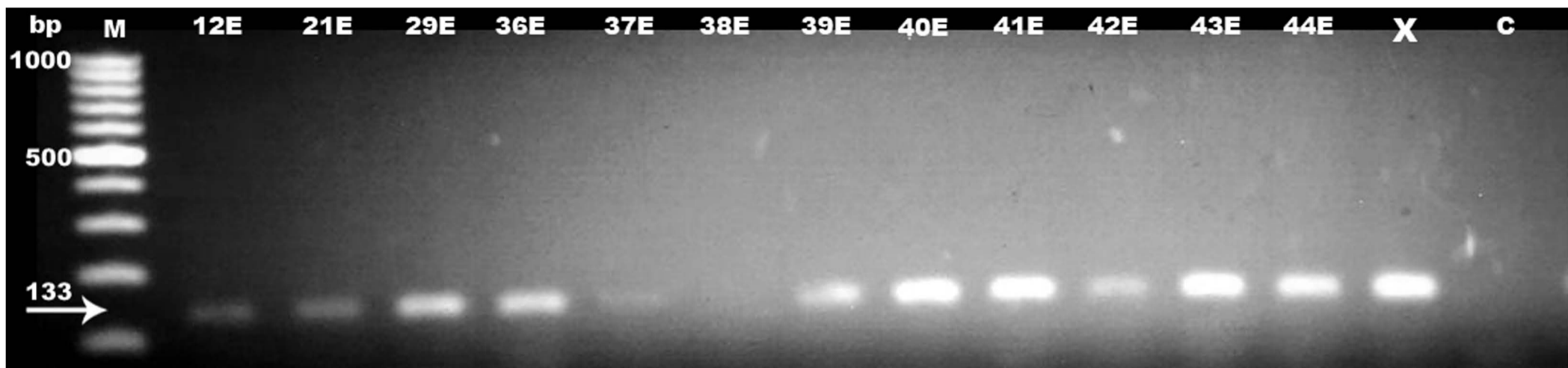

**Fig. (3) A**

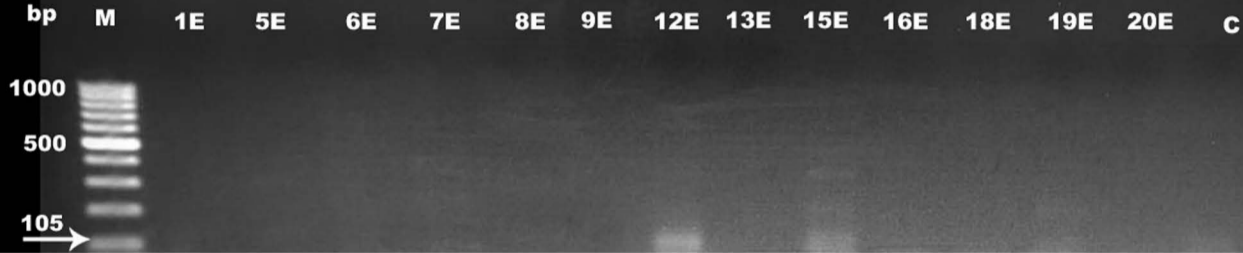

**Fig. (3) B**

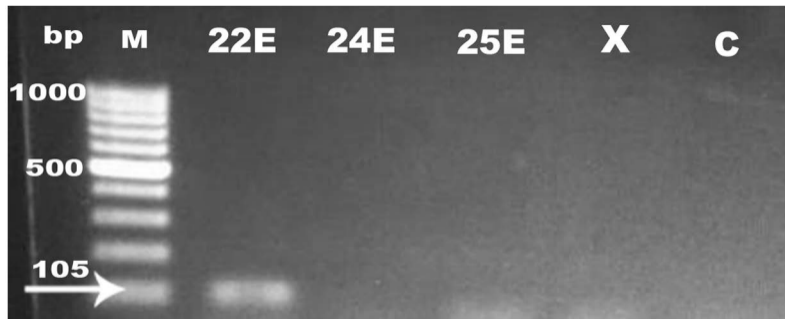

**Fig. (3) B**

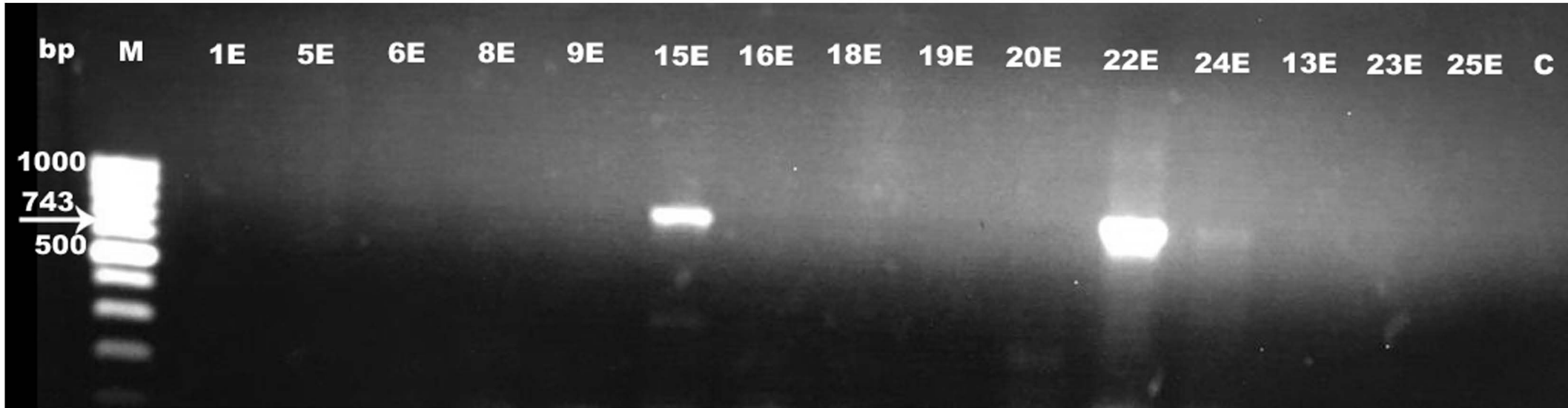

**Fig. (3) C**

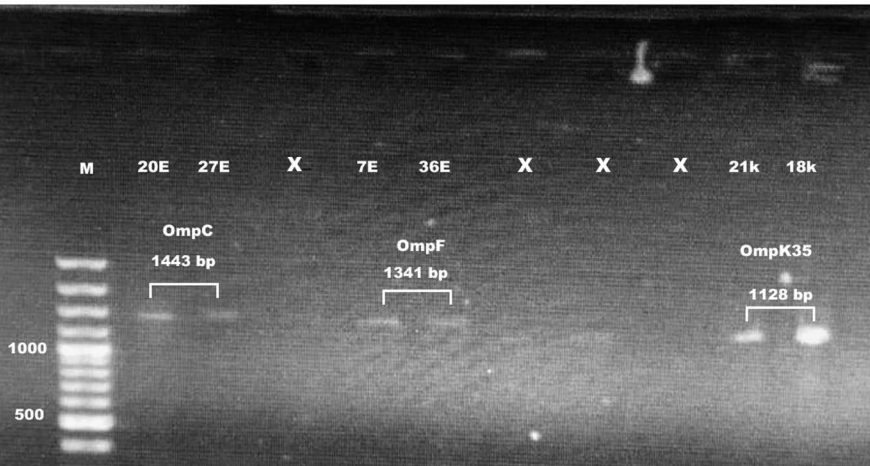

**Fig. (6) A**

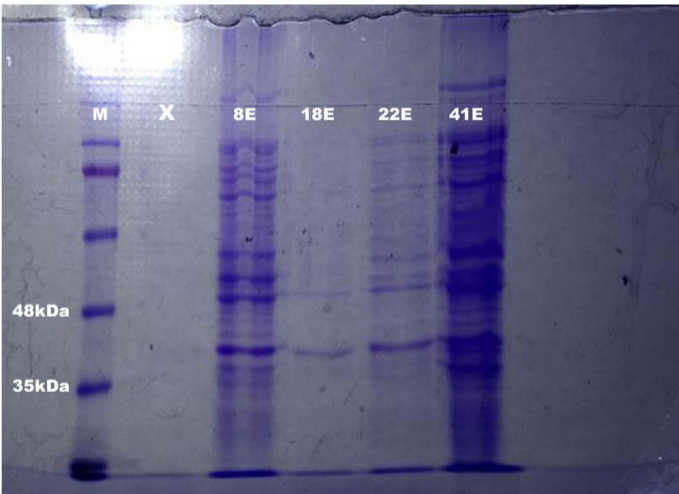

**Fig. (6) B**

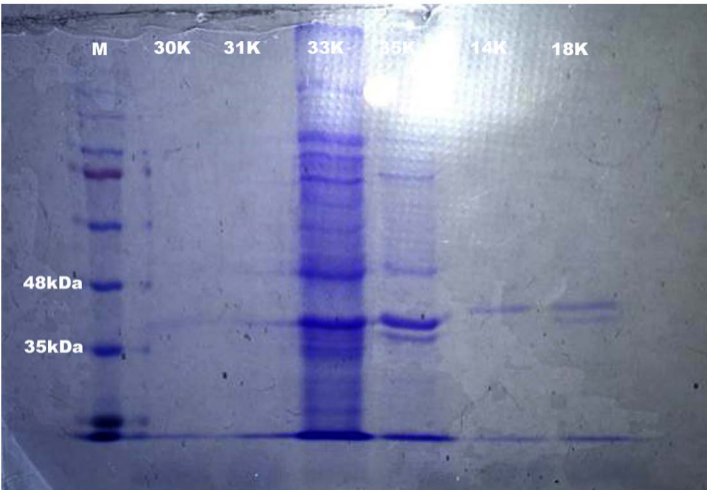

**Fig. (6) C**

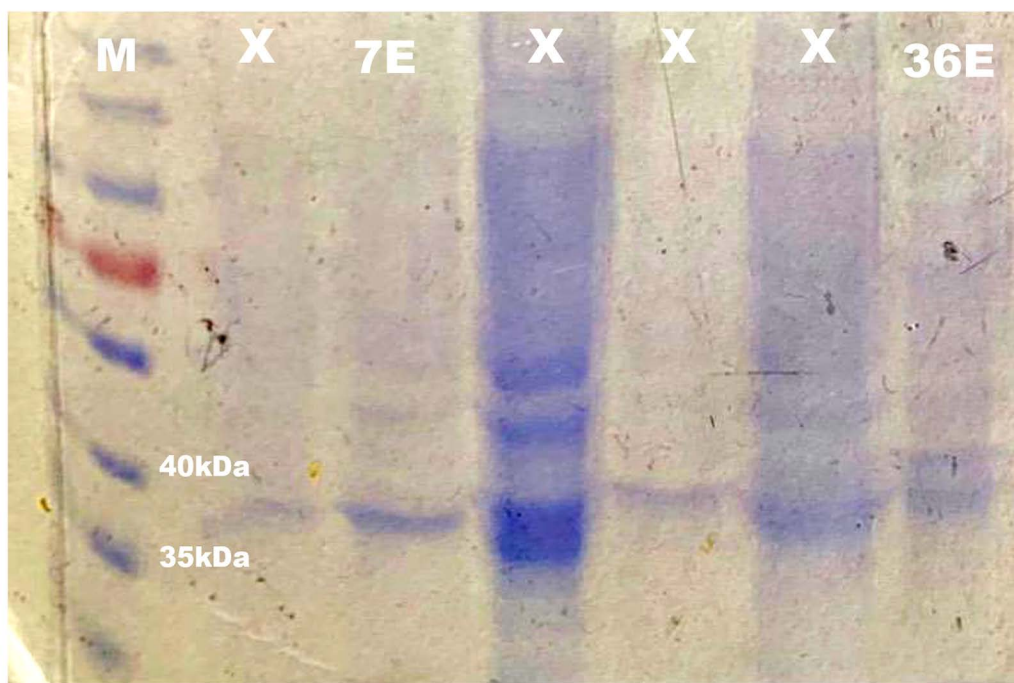

**Fig. (7) A**

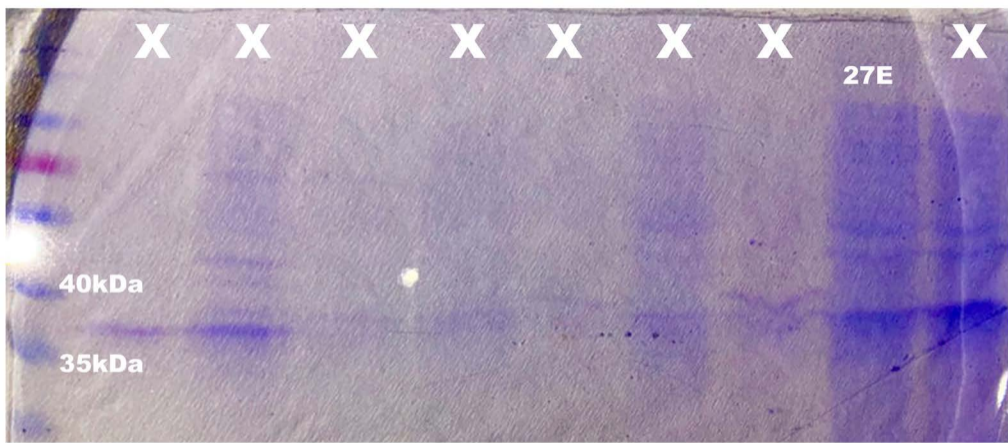

**Fig. (7) A**

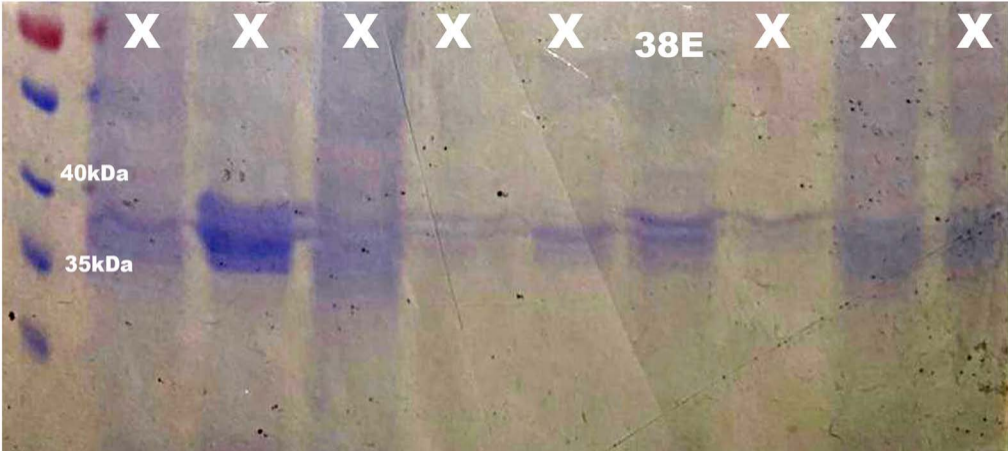

**Fig. (7) A**

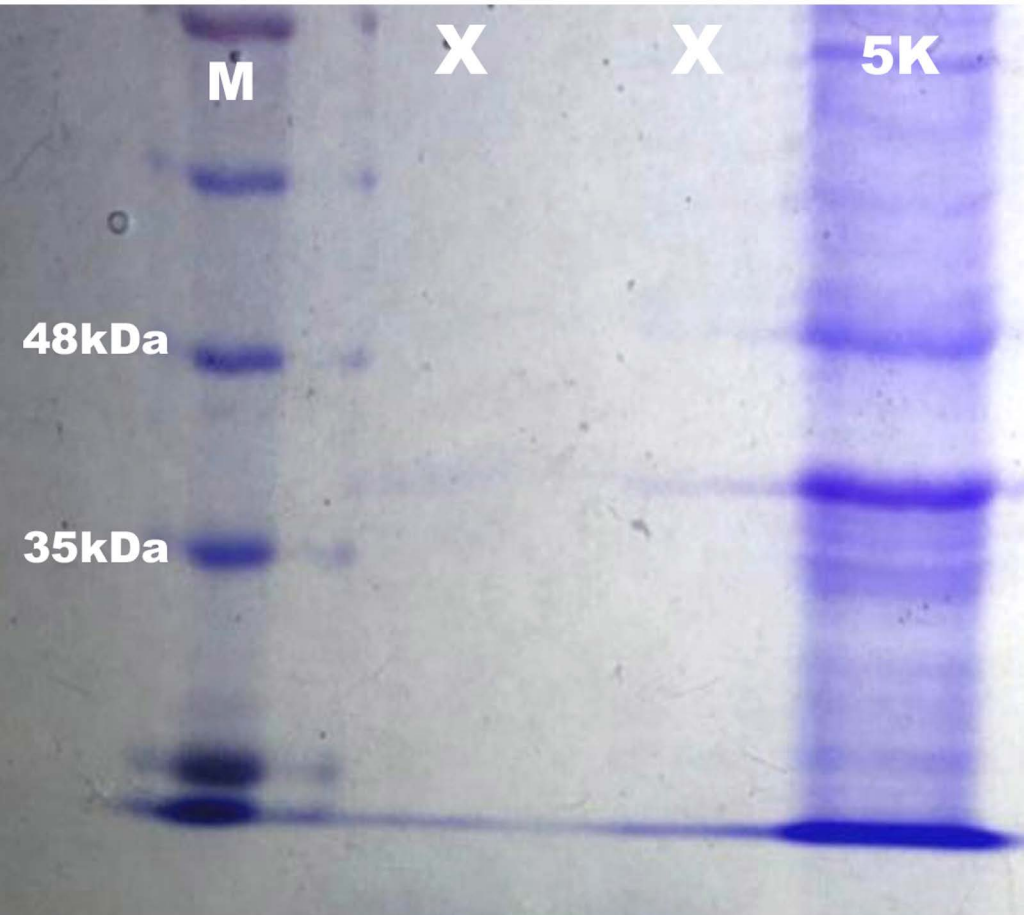

**Fig. (7) B**

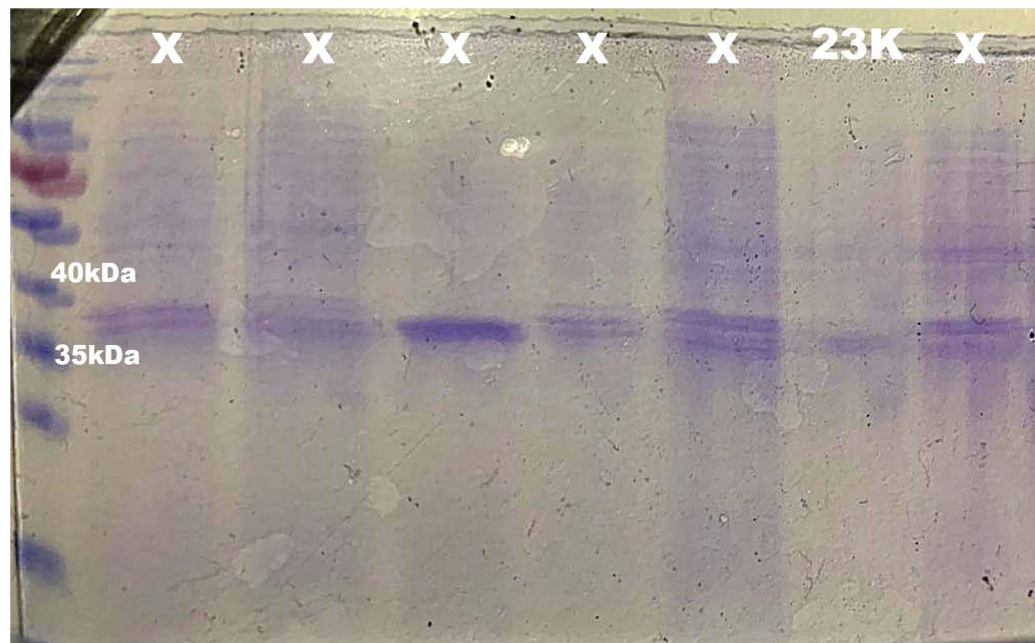

**Fig. (7) B**

Supplement: S1 File — (PDF) [file pone.0251594.s003.pdf]
